# Barriers and Enablers to Joining the National Organ Donation Registry Among Patient Population at a Tertiary Care Hospital of Peshawar, Pakistan

**DOI:** 10.7759/cureus.37997

**Published:** 2023-04-22

**Authors:** Yusra Samin, Talha Durrani, Aima Yousaf, Manahil Majid, Dawood Misbah, Maimoona Zahoor, Muhammad Adeel Khan

**Affiliations:** 1 General Medicine, Khyber Teaching Hospital, Peshawar, PAK; 2 General Medicine, Lady Reading Hospital, Peshawar, PAK; 3 Medical Oncology, Shaukat Khanam Hospital, Peshawar, PAK; 4 General Medicine, Rehman Medical Institute, Peshawar, PAK

**Keywords:** organ donation registries, patient attitudes, brain death, ogran transplant, organ donation

## Abstract

Background

Organ transplantation is a life-saving therapy for patients with end-stage organ failure. However, the demand for organs far exceeds their availability, leading to longer waiting times and increased mortality rates. Pakistan faces a similar situation, with a shortage of organ donors and several barriers to therapeutic organ donation, including cultural, religious, and political ones.

Objective

The objective of this study was to understand the barriers and enablers to joining the national organ donation registry among patient populations at a tertiary care hospital in Peshawar, Pakistan. The findings can then guide targeted educational campaigns to improve the state of therapeutic organ transplants in the country.

Methods

A descriptive, cross-sectional study was conducted at the Outpatient Departments of Lady Reading Hospital, Peshawar, targeting all patients and visitors aged 18 to 60 who presented to the outpatient departments of the hospital. A modified and validated questionnaire was used to collect data, which were analyzed using Statistical Package for Social Sciences (SPSS) version 26.

Results:

The study analyzed the attitudes of 342 individuals, among which 82.18% had not heard about Pakistan's Organ Donation Registry, 58.09% agreed with organ donation, and 23.68% suggested they would like to join the registry someday. Religious beliefs and the lack of knowledge about the laws related to organ donation stood out as statistically significant barriers to joining the national organ donation registry of Pakistan (p<0.05). The study also found that the willingness to donate was significantly higher among those who themselves encouraged organ donation and were willing to do so if the country's system were to support it (p<0.05).

Conclusion

The majority of participants had not heard of the organ donation registry, and a lack of knowledge about the legal framework and religious beliefs were significant barriers to joining the registry. This is hindering the growth of therapeutic organ transplantation in Pakistan. In addition, the willingness to donate was higher among those who supported organ donation and believed in its benefits. Increasing awareness and promoting a culture of organ donation in Pakistan can help address the shortage of organ donors and improve the state of therapeutic organ transplantation in the country.

## Introduction

End-stage organ failure is the leading cause of mortality in patients admitted to the intensive care unit (ICU), with high demand for organs such as heart, lungs, kidneys, and liver [1]. Organ transplantation is the only viable option for many critically ill patients suffering from end-stage organ failure [2]. The field of organ transplant has expanded significantly over the last few decades, with advances in immunology, immunosuppression, and surgery. Despite these advancements, the availability of organs for transplantation is inadequate to meet the rising demand worldwide, even in countries such as the United States, Canada, and Australia [3-5]. This shortage of organs has led to longer waiting times and increased mortality rates among patients needing organ transplantation. A lack of organ donors is a global problem with known barriers, including cultural, religious, and political ones [6]. Pakistan, one of the largest commercial suppliers of kidneys for transplant, faces a similar situation where the demand for organ donation far surpasses its availability [7]. In Pakistan, an estimated 50,000 people die yearly due to end-stage organ failure. This figure includes 10,000 with liver failure and 6500 with heart failure [8]. Approximately 15,000 people die of kidney failure, but there are only 1500 kidney transplants in a year. Moreover, the waiting list for liver transplantation in Pakistan is also quite long, with around 8,000 to 10,000 people waiting for a liver transplant, and only around 200 liver transplants are performed each year in the country [9]. 

There are several barriers to therapeutic organ donation in Pakistan, including the sale of organs for entrepreneurial reasons, religious concerns, and a lack of public awareness [10, 11].

Understanding the barriers and enablers to joining the national organ donation registry is crucial for developing effective educational campaigns encouraging the public to donate organs. Most organ donation surveys conducted in Pakistan have focused on the attitudes of the general public, but there has been little published attention to the differences between potential organ donors and non-donors, which addresses a more specific and potentially more significant research gap. Focusing on these groups will likely provide valuable information that can guide targeted educational campaigns to improve the state of therapeutic organ transplants in the country.

## Materials and methods

We conducted a descriptive, cross-sectional study at the Outpatient Departments of Lady Reading Hospital, Peshawar, from October 2021 to March 2022, for a total of 26 weeks. Ethical approval was obtained from the Institutional Review Board (IRB) at Lady Reading Hospital, Peshawar, under reference number 53/LRH/MTI, on 18-02-2021. The sample size of 350 was determined based on a previously published study [12]. We used a convenience sampling technique for this survey, targeting all patients and visitors aged 18 to 60, regardless of gender, who presented to the outpatient departments of the hospital.

Recruitment criteria

We used the following recruitment criteria: 1) adult patients aged 18 to 60 years, irrespective of gender; 2) patients who were willing to respond, while those who were unwilling or in critical condition were excluded from the study.

Questionnaire

We used a modified version of an already validated questionnaire from a previously published study and added items regarding recurring themes in relevant studies [12]. The questionnaire was divided into two parts: (1) the sociodemographic characteristics of the participants and (2) their attitudes toward organ donation. Brief instructions and explanations were provided for each section. The English version of the questionnaire was translated into Urdu, the national language of Pakistan, with the help of a Pakistani expert. The translated version was back-translated and evaluated by an independent researcher to ensure cultural appropriateness. The Urdu version was pre-tested with 16 patients. The final version was evaluated for internal reliability, which was calculated using Cronbach's alpha, yielding a value >0.92. The final version was used for data collection. We used Statistical Package for the Social Sciences (SPSS; IMB Inc., Armonk, US) version 26 for data analysis. All categorical variables were reported as frequencies and percentages, while continuous variables were reported as means ± standard deviation (SD). Chi-square and independent Student's t-tests were used for data analysis. A p-value of <0.05 was considered statistically significant.

## Results

Out of the initial sample size of 350 patients, eight patients did not complete the questionnaire, resulting in a final analysis of 342 individuals. The demographic characteristics (Table 1) of the sample population were as follows: the mean age was 31.17 years, 65.02% were female, 80.70% lived in rural areas, 71.62% were married, and 42.69% had not attended formal schooling. In terms of employment, 65.50% were employed, and 33.33% had a chronic disease.

**Table 1:** Demographic characteristics

Variable		n	%
Age, years (mean ∓ SD)		31.17 ∓ 7.88	
Gender	Male	120	34.98
	Female	222	65.02
Residence	Urban	66	19.30
	Rural	276	80.70
Marital status	Unmarried	97	28.36
	Married	245	71.62
Level of education	No formal schooling	146	42.69
	Secondary school	125	36.55
	College	26	7.59
	University	45	13.16
Current job	Private work	35	10.23
	Employed	224	65.50
	Unemployed	83	24.27
Chronic disease	Present	114	33.33
	Absent	228	66.67

When asked about their attitudes towards organ donation, 82.18% had not heard about Pakistan's Organ Donation Registry, 58.09% agreed with organ donation, and 23.68% would like to join the registry someday (Table 2). In addition, 54.39% would agree to donate the organs of a family member after their death, and 70.47% would donate if religion and law encouraged it. The study also analyzed the relationship between demographic characteristics and willingness to donate. The results showed that those who lived in urban areas (p=0.873) and those with chronic diseases (p=0.284) were not significantly different in their willingness to donate. However, there was a significant difference in willingness to donate between those who encouraged organ donation (p<0.05) and those who did not, those who would donate if the law encouraged it (p<0.05), and those who were aware of the laws related to organ donation (p<0.05).

**Table 2:** Factors influencing willingness to join the National Organ Donation Registry

Variable		Willingness to join the National Organ Donation Registry		Total	p-value
		Yes	No		
Age, years (mean ∓ SD)		34.6	32.5		0.115
Residence	Urban	16	50	66	0.873
	Rural	65	211	276	
Marital status	Unmarried	23	74	97	1
	Married	58	187	245	
Education	Illiterate	42	106	148	0.95
	Some formal education	39	155	194	
Employment	Employed	61	198	259	1
	Unemployed	20	63	83	
Chronic disease	Present	31	83	114	0.284
	Absent	50	178	228	
Have you ever heard of organ donation?	Yes	61	220	281	0.7
	No	20	41	61	
Would you encourage organ donation?	Yes	72	154	226	<0.05
	No	3	113	116	
If religion were encouraging organ donation, would you, do it?	Yes	65	214	279	0.7
	No	26	37	63	
If the law of the country were encouraging organ donation, would you, do it?	Yes	76	163	241	<0.05
	No	5	98	101	
Are you aware of the laws and legislations related to organ donation transplantation in Pakistan?	Yes	25	19	44	<0.05
	No	56	242	298	
Would you agree to donate the organs of a family member after their death?	Yes	27	61	88	0.08
	No	54	200	254	
If you were to donate after death, who would you want to donate your organs to?	Family only	14	65	79	0.176
	Anyone in need	67	196	263	

Regarding the reasons for agreeing or disagreeing to donate organs, 80 individuals disagreed due to religious beliefs, while 137 disagreed, citing that the recipients were not chosen fairly (Table 3). When asked about their motivations for donating organs, 264 individuals responded that they wanted to help, and 147 cited religious reasons.

**Table 3:** Public perceptions and attitudes toward organ donation

Variable		n
Where did you hear about organ donation?	Social media	87
	Television	187
	Newspapers and magazines	29
	Internet	87
	Educational center	19
	Friends and family	99
	Health-care workers	23
As far as you are concerned, what do you relate organ donation to?	Donation after death	211
	Donation during life	181
	Brain death	58
	Organ trafficking	59
In case you disagreed to donate your organs, what would the reasons be?	Absence of financial benefit	6
	Religious beliefs	80
	Social and familial barriers	99
	Fear of not receiving adequate medical care	117
	Fear of talking about death	33
	Lack of knowledge about organ donation	85
	Organ recipients are not chosen fairly	137
In case you agreed to donate your organs, what would your motivations be?	Religious	147
	It does not harm, so why not?	141
	The desire to help/altruism	264
	Financial	6

## Discussion

Organ transplantation is considered one of the greatest advances in modern medicine. It provides a ray of hope and the sole therapy for many organ insufficiencies. However, there is always a gap between organ donation and its supply, even in first-world countries like the United States. Several factors, including education status, socioeconomic status, religious beliefs, family, and social attitudes, affect organ donation [13].

This study aimed to identify the barriers and enablers to joining the National Organ Donation Registry among patients at a tertiary care hospital in Pakistan. Our findings indicate that a high percentage of patients (82.18%) were aware of organ donation, which is comparable to previous studies conducted in Pakistan, showing between 60-85%. However, only 26.23% of the study population expressed a willingness to donate an organ, indicating a significant gap between awareness and actual donation rates [14-16]. The difference can be explained by the fact that all these studies were conducted between 2005 and 2009, after which a lot of awareness and education work has been done to clear ambiguities regarding organ donation in the general population [17]. However, there is a lot of space and a need for further improvement.

Religious beliefs and lack of knowledge about the laws and regulations related to organ donation were identified as barriers to organ donation in our study. Regarding religious beliefs, the majority of the Pakistani population is Muslim, and while several Fatwas have been published stating that organ donation is morally permissible in Islam, religious beliefs still pose a barrier to organ donation. Our study revealed a significant association between unwillingness to donate organs and religious belief and unawareness of the laws and legislations related to organ donation transplantation in Pakistan (p≤0.05), a similar trend that was observed in a study conducted in the United Kingdom among university students of Indian and Pakistani descent showed the same significant association of organ donation with religious belief [18]. Thus, there is a need to educate the general population about the permissibility of organ donation in Islam and clarify any ambiguities about organ donation and transplantation.

In addition to religious beliefs, a lack of knowledge about brain death was identified as a barrier to organ donation in our study. Our study revealed that 61.6% of the target population knew that organs could be donated after death, and 52.92% knew that organs could also be donated during life. These findings can be compared to another study done in Pakistan in which the percentage was 50.1% and 36.5%, respectively [15]. However, only 16.9% of the study population knew about brain death, indicating a lack of awareness about the medical concepts related to organ donation. To increase organ donation rates, there is a need to educate the general population about brain death and its significance in organ donation and transplantation. Our study found that only 26.23% of the study population agreed to join the National Registry and have an organ donated after death, which was similar to the findings of neighboring countries like Western India, and China, in which only 16% and 49.8% of the study population agreed to donate organs, respectively [19,20].

These findings point toward the need for education regarding the permission of organ donation in Islam and to give adequate knowledge about the laws related to organ donation in Pakistan [12,21]. However, only 29.25% of the study population agreed to attend a seminar about organ donation. This could be because of a lack of interest among the general population in organ donation. This shows the need for time to intervene and raise awareness about organ donation and transplantation to increase the number of potential donors.

Despite the useful findings, there are some limitations to this study. Firstly, understanding the nuanced experiences and perspectives of patients is better done by an open-ended questionnaire and a qualitative study design. However, we proceeded with a quantitative study design, with themes included from previously published studies, to achieve a higher sample size and robust statistical analysis. Secondly, the study was conducted at a single tertiary care hospital in Pakistan, which may limit the generalizability of the results to other settings in the country. Furthermore, the study used a convenience sampling technique, which may have resulted in selection bias and affected the representativeness of the sample.

## Conclusions

Religious beliefs, lack of knowledge about the laws and regulations related to organ donation, and lack of awareness about brain death are significant barriers to joining the National Organ Donation Registry in Pakistan. Our findings emphasize the importance of educating the general population about organ donation, brain death, and legal and religious perspectives on the issue. It also underscores the need to involve healthcare professionals in awareness campaigns and training medical students to address the issue of organ shortage effectively.
